# 

**Published:** 1872-09

**Authors:** 


### ARTICLE IV.

### *American Academy of Dental Science.*

The Fifth Annual Meeting of the American Academy of Dental Science, will be held in Boston on Monday, September 30th, 1872, at eleven o'clock A. M.

The Annual address, will be delivered by Prof. P. H. Austen, of Baltimore.

E. N. HARRIS, D. D. S. Cor'g. Sec'y.,
Boston, Mass.

